# Supplementary material for: Elp1 is required for development of visceral sensory peripheral and central circuitry
Source: Dis Model Mech. 2022 Jun 1;15(5):dmm049274. doi: 10.1242/dmm.049274 (PMC9187870; doi:10.1242/dmm.049274)
Supplement: Supplementary information [file dmm-15-049274-s1.pdf]

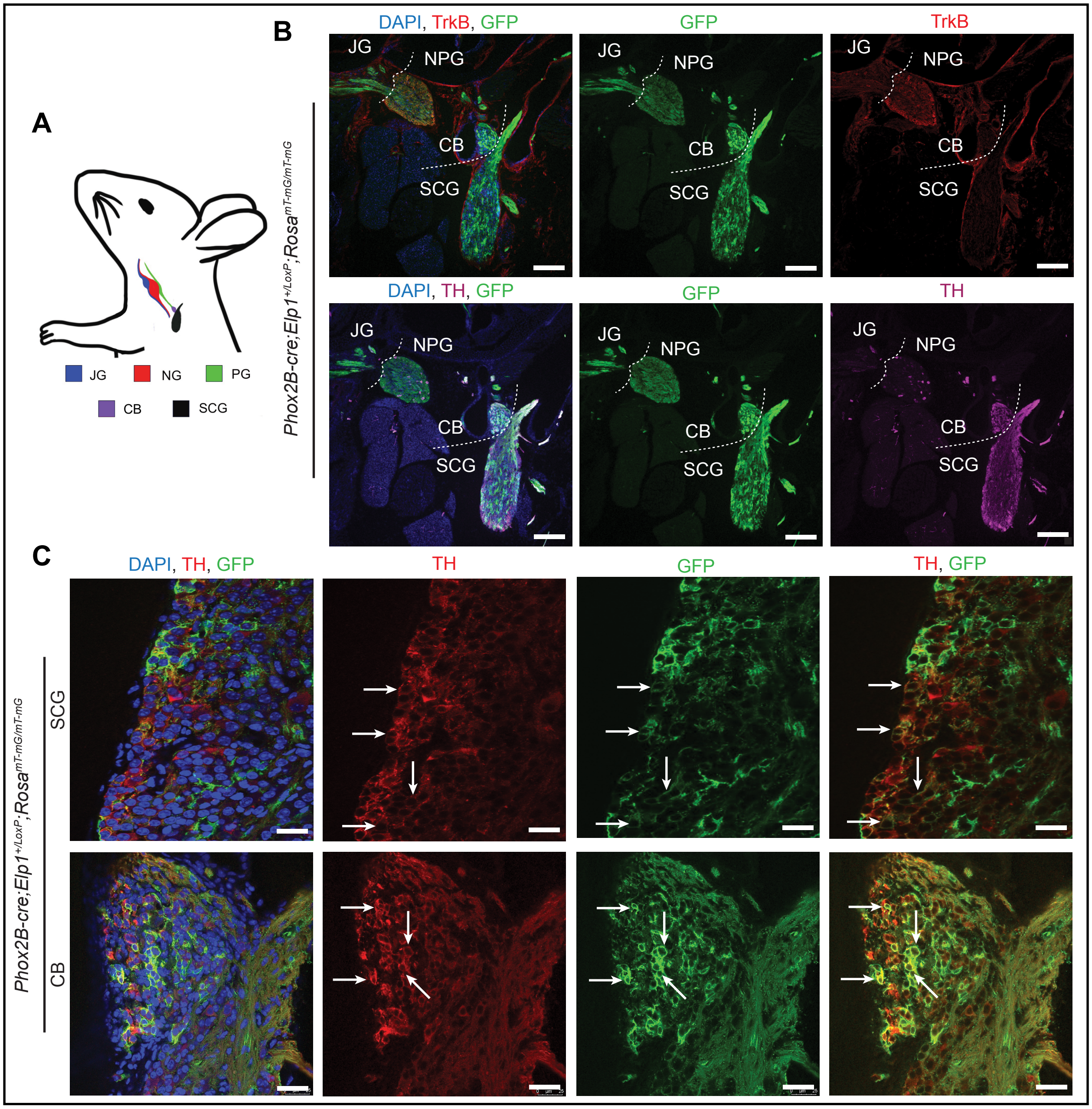

Disease Models & Mechanisms • Supplementary information

Fig. S1. Low magnification view of location of visceral cranial ganglia IX and X, carotid body and superior cervical ganglion and expression of the *Phox2b-cre*. A schematic of the locations of the jugular ganglion (JG, blue), nodose ganglion (NG, red), petrosal ganglion (PG, green), carotid body (CB, purple), and superior cervical ganglion (SCG, black) is shown (A). Mice that are heterozygous for *Elp1*^{loxp} (i.e. express no mutant phenotype because they have one normal copy of *Elp1*) and express *Phox2b-cre* and *Rosa*$^{mT\text{-}mG/mT\text{-}mG}$ were sectioned at P1 and stained with antibodies to TrkB and TH. Careful analysis of Phox2b-cre expression using a Rosa26 mTmG reporter show that the Phox2b-cre is expressed throughout the placode-derived nodose and petrosal ganglia, and not in the neural-crest derived jugular ganglion consistent with the report of Rossi et al. (2011).The sagittal sections depicted in (B) show that the JG is GFP- and TrkB-, the NG is GFP+ and TrkB+, while the CB and SCG are TrkB- but do contain some GFP+ cells, meaning that some cells in the CB and in the SCG do express the *Phox2b-cre*. GFP+ NP axons can be detected running through the JG, but the neurons in the JG itself do not express GFP (lower panels). White dotted lines indicate separations of ganglia or separation of CB from SCG. (C) Colocalization of TH and GFP in the CB and SCG are shown with white arrows pointing to cells that co-express TH and GFP. N = 2 mice. Abbreviations: CB, carotid body; PG, petrosal ganglion; NP, nodose-petrosal ganglia; SCG, superior cervical ganglion; NG, nodose ganglion; JG, jugular ganglion. Scale bar = 250 microns (B); 25 microns (C).

Disease Models & Mechanisms • Supplementary information

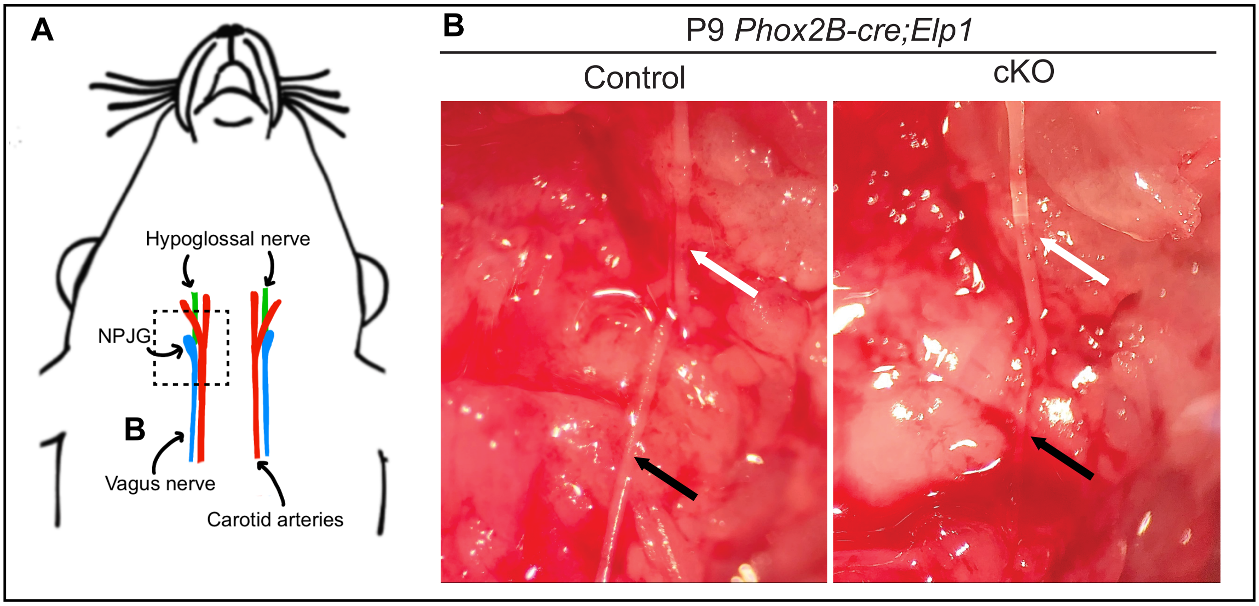

Fig. S2. Vagus nerve is reduced in diameter in ***Elp1*** cKO mice compared to control littermates. (A) A schematic identifying the location for analysis of vagus and hypoglossial nerves in (B). *Phox2b-cre;Elp1* control and cKO mice at P9 were dissected, and carotid arteries removed to reveal their vagus nerves (black arrows). Their hypoglossal nerves (indicated by the white arrow) are comparable in diameter between the cKO and control, but the vagus nerve in the cKO mouse is smaller in diameter than it is in the control mouse.

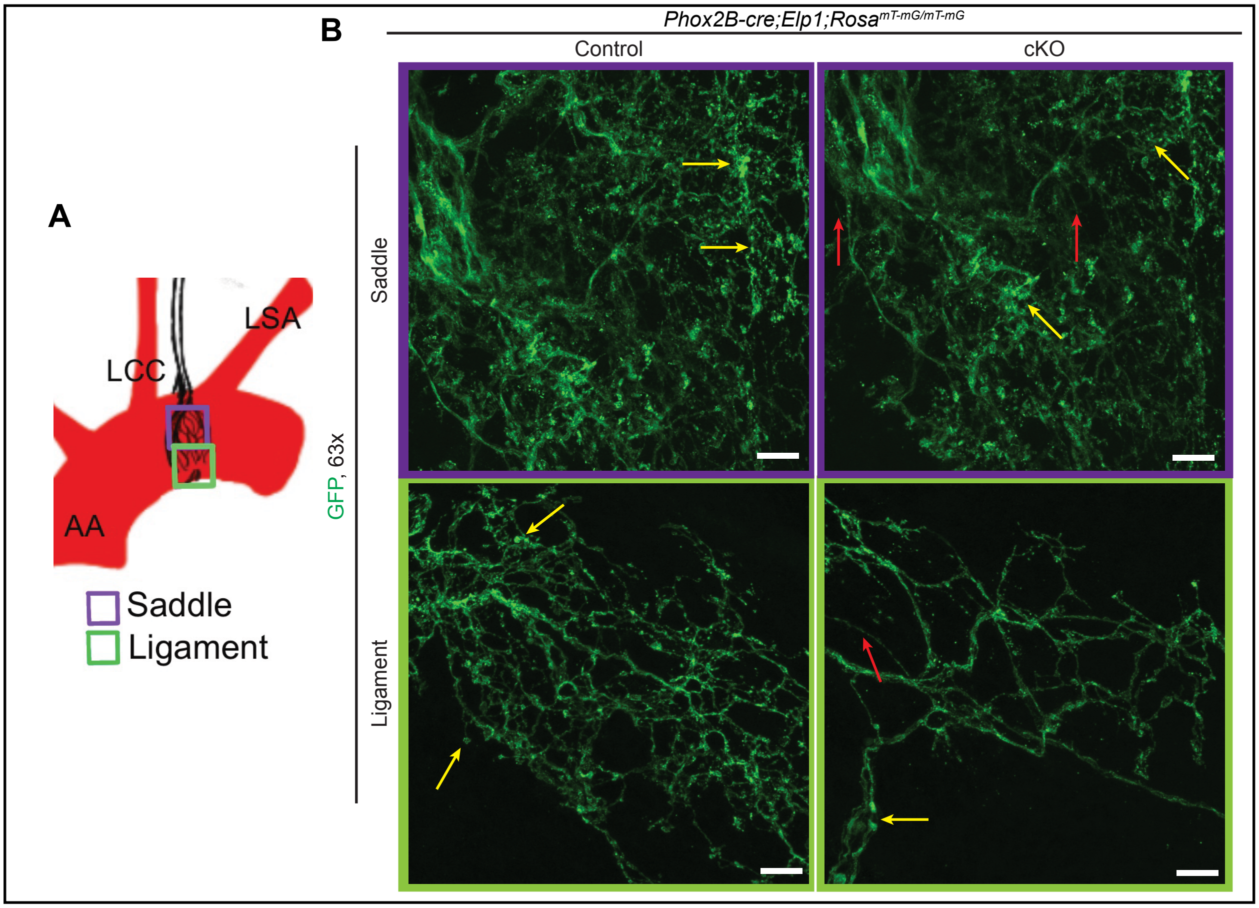

Fig. S3. Elp1 is required for normal end-net endings and flower-spray innervation of the aortic arch. (A) Schematic depicting the locations of the left baroreceptors, with the saddle (purple) and the ligament (green) indicated. End-net endings (red arrows) and flower spray endings (yellow arrows) for the baroreceptors in the saddle (purple border, upper panels) and the ligament (green border, lower panels) of cKO *Phox2b;Elp1*$^{LoxP/LoxP}$*;Rosa*$^{mT\text{-}mG/mT\text{-}mG}$ mice are reduced compared to control *Phox2b;Elp1*$^{+/LoxP}$*;Rosa*$^{mT\text{-}mG/mT\text{-}mG}$ mice (B). N=4 mice from each condition. Abbreviations: AA: Aortic Arch, LCC: left common carotid, LSA: left subclavian artery. Scale bar = 25 microns.

Disease Models & Mechanisms • Supplementary information
